# Exercise-induced ventricular tachycardia in a young adult: what lies beneath?

**DOI:** 10.1007/s12471-026-02043-9

**Published:** 2026-04-10

**Authors:** Ana Sofia Nogueira Fernandes, Rui Pedro Files Flores, Sérgia Andreia Alves Rodrigues da Rocha Costa

**Affiliations:** Department of Cardiology, ULS Braga, Braga, Portugal

## Anwser

The electrocardiographic findings provided important diagnostic clues. The ECG during tachycardia demonstrated a wide-complex rhythm with left bundle branch block morphology, superior axis, and late R‑wave transition in the precordial leads—a pattern suggesting with a sustained monomorphic ventricular tachycardia (SMVT) originating from the right ventricle (RV), most likely the inferoapical region. The ECG in sinus rhythm revealed T‑wave inversion in the inferior and lateral precordial leads, suggesting underlying structural abnormalities.

**Fig. 1:**
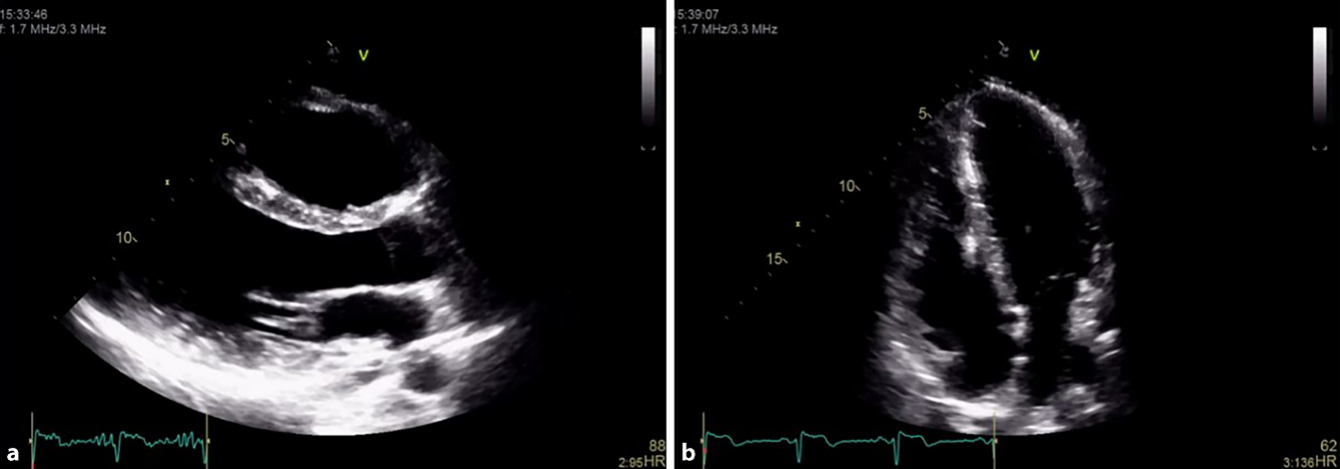
Transthoracic echocardiogram demonstrating right ventricular dilation and systolic dysfunction

**Fig. 2:**
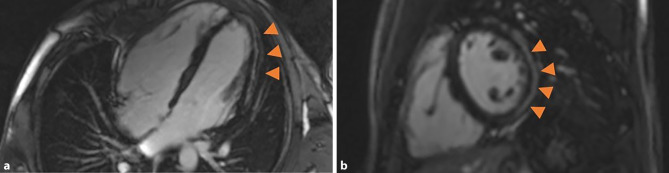
Cardiac magnetic resonance imaging. Four-chamber view (**a**) and short-axis view (**b**) demonstrate right ventricular dilation and subepicardial late gadolinium enhancement (arrows) involving the mid-apical inferolateral and anterolateral walls

Transthoracic echocardiography showed a dilated and dysfunctional RV (Fig. 1). Cardiac magnetic resonance confirmed RV dilation with systolic dysfunction (RVEF 37%) and apical hypokinesia. The left ventricle was nondilated with normal systolic function (LVEF 55%) and subepicardial fibrosis in the mid-apical inferolateral and anterolateral walls (Fig. 2). Genetic testing identified a splice-site likely pathogenic variant in the PKP2 gene, the most frequently implicated desmosomal gene in arrhythmogenic cardiomyopathy (ACM).

According to the 2024 European Task Force criteria [1], the patient met three major (RV dilation/dysfunction; left ventricular non-ischemic fibrosis; SMVT from the right ventricular) and two minor (T-wave inversion in the inferior and left precordial leads; likely pathogenic PKP2 variant) criteria from different categories, consistent with definite biventricular ACM.

Given the life-threatening arrhythmia and underlying structural abnormalities, a single-chamber transvenous implantable cardioverter-defibrillator was implanted for secondary prevention, enabling antitachycardia pacing to terminate SMVT and reduce shocks. The patient remained asymptomatic on beta-blocker therapy during follow-up, without recurrence of ventricular arrhythmias [2].

This case highlights the complementary role of ECG, imaging, genetics, and updated diagnostic criteria in confirming ACM and optimizing risk stratification.

## Data Availability

The data supporting the findings of this study are available from the corresponding author upon reasonable request, in accordance with patient confidentiality and institutional regulations.
